# Machine Learning-Enhanced Dual-Band Plasmonic Sensing for Simultaneous Qualitative and Quantitative Detection of Biomolecules in the Mid-Infrared Region

**DOI:** 10.3390/s25103135

**Published:** 2025-05-15

**Authors:** Yunwei Chang, Ang Bian

**Affiliations:** Department of Optoelectronic Information Science and Engineering, School of Science, Jiangsu University of Science and Technology, Zhenjiang 212100, China; angbian@just.edu.cn

**Keywords:** plasmon, molecular sensing, machine learning algorithm

## Abstract

Recently, sensing for biomolecules has become increasingly popular in the fields of environmental monitoring, personal health, and food safety. Plasmonic biosensors have been a powerful tool due to their high sensitivity and label-free operation. However, when it comes to molecules with different kinds and concentrations, detection technology and data processing remain a challenging task. In this study, we investigate the qualitative and quantitative detection of two kinds of biomolecules in the mid-infrared region simultaneously by the utilization of a plasmonic sensor. The strong coupling between each plasmonic resonance and the corresponding molecular vibration is found to significantly enhance the absorption signal of molecules, and the obtained Rabi splitting is not only a proof of molecular existence but also an indicator of molecular concentration. However, the amount of the molecular solution with a background refractive index in turn affects the plasmonic resonance position. In more general situations, it is not easy to achieve the match between plasmonic resonance and molecular resonance, and thus the quantitative detection by the Rabi splitting depth is not always feasible. Hence, we propose a machine learning algorithm called principal component analysis (PCA), providing a versatile approach for analyzing the proportion of each molecule in the mixture. Our work opens up new routes in noninvasive optical sensing and the integration of AI-driven data analysis further strengthens its potential for real-world applications.

## 1. Introduction

Nowadays, the detection of infrared molecular vibrations is of vital importance as they provide unique information about specific biomolecules including proteins, lipids, nucleic acids, glucose, and DNA [1,2], which has significant implications in a wide range of fields, such as chemical sensing, pharmaceutical research, environmental monitoring, and personal health [3,4,5]. However, due to the intrinsically low absorption cross-sections, the signal of molecular vibration itself is weak and challenges the detection process [6]. Owing to the advantages of the localized and enhanced electromagnetic field (hot spots), surface plasmon resonance (SPR) has been implemented as a promising approach to overcome this constraint [7,8]. Through the strong interaction between the vibrational modes of molecules and the local electromagnetic fields of plasmonic resonators, the signals of molecules can be apparently amplified, realizing surface-enhanced infrared absorption (SEIRA) [9,10,11]. This effect, furthermore, is more effective when molecules are located to have spatial overlap with the hot spot as much as possible.

Generally, an MDM (metal–dielectric–metal) structure is the commonly used plasmonic sensor [12,13,14], which consists of metallic antennas on top of a metallic layer separated by a thin dielectric spacer layer. The highly enhanced field is partly concentrated around the top metallic antennas, and more is highly confined in the sandwiched thin insulating layer. The targeted biochemical molecules spread on the metallic antennas have access to interact with the surrounding enhanced field, while another portion of the enhanced nearfields in the dielectric layer is inaccessible to the molecules. Hence, recent improvements have been made to provide an increased sensing area for the target molecules [15,16,17,18]. Hwang et al. used gold (Au) cross-shaped antennas on dielectric pedestals, which are etched into cross-shaped antennas with a size smaller than the metal antennas, providing an increased effective sensing area for molecules [15]. On the other hand, a conventional antenna, exhibiting a resonance, cannot simultaneously operate in two bands. When one wants to identify more than one molecular fingerprint simultaneously, dual or multiple plasmonic resonances will be more desirable.

When the resonance position of the plasmon is tuned to match with a certain molecular vibration band, the strong coupling arises and leads to the Rabi splitting, which can be the direct evidence of the presence of the corresponding molecule in the mixed analytes [19,20,21]. When it comes to the quantitative detection in molecular mixed solutions, it is reported that the amount of the molecular solution with a background refractive index in turn affects the plasmonic resonance position [19,20,21], which is unable to ensure the perpetual alignment match between plasmonic and molecular resonance. Hence, it is infeasible to identify the concentration ratio of individual molecules directly by the naked eye. A mathematical algorithm known as principal component analysis (PCA) can be used for state-of-the-art data analysis [22,23]. Apart from its intrinsic stability against dynamic changes in the sensor environment, the algorithm is independent of input parameters and thus, completely objective and fully autonomous. Hence, the combination of SEIRA and PCA is uniquely suited for a new noninvasive sensor principle.

In this paper, we investigate the qualitative and quantitative detection of multiple molecular vibrations in the mid-infrared region by the utilization of a dual-band plasmonic absorber (DBPA), which consists of an asymmetric cross-shaped Au nanoantenna array on an etched cross-shaped MgF2 dielectric spacer, and a thick Au mirror layer. Each plasmonic resonance wavelength can be independently tuned to overlap with the wavelength of vibrational fingerprints in the mixing analytes by adjusting the length of the Au cross antenna. The strong coupling between each plasmonic resonance and the corresponding molecular vibration is expected to significantly enhance the absorption signal of molecules, and the obtained Rabi splitting can manifest the presence of a specific molecular vibration. Moreover, we propose a versatile method—principal component analysis (PCA)—to detect the corresponding concentration quantitatively.

## 2. Materials and Methods

**Figure 1:**
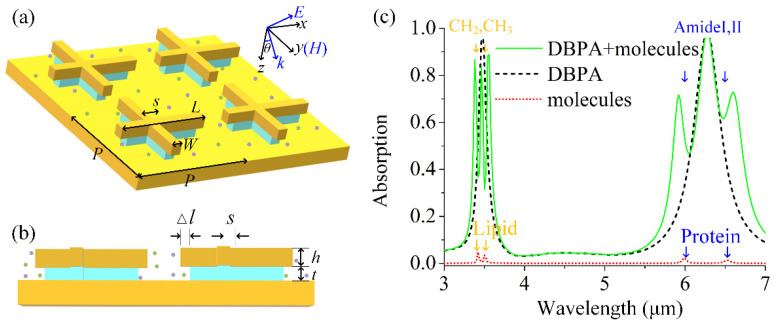
(**a**) Schematic of the dual-band plasmonic absorber (DBPA): asymmetric cross-shaped Au nanoantenna array (length *L*_1_ = 1.33 µm, width *W* = 0.24 µm, asymmetric parameter *s* = 0.265 um, and period *P* = 1.6 µm) on a MgF_2_ dielectric spacer and Au substrate. (**b**) Side view of DBPA structure with top Au layer *h* = 70 nm and MgF_2_ dielectric spacer *t* = 90 nm. The size of the MgF_2_ cross-shaped structure is Δ*l* smaller than that of the Au nanoantenna. (**c**) Absorption spectra of the bare DBPA (dashed line) and bare molecules without the assistance of plasmon (dotted line), and the coupled DBPA with molecules covering it (solid line). Molecules include lipid (CH_2_ 3427/CH_3_ 3509 nm) and protein (Amide I 6020/Amide П 6410 nm) vibrational bands and show amplified molecular signals after coupling.

Figure 1a illustrates the schematic view of the dual-band perfect absorber (DBPA) design under study. An asymmetrical cross-shaped Au nanoantenna array was placed on top of a thick Au mirror layer, separated by a MgF_2_ dielectric isomorphic cross-shaped spacer with a size slightly smaller than the top Au nanoantenna. The cross-shaped Au nanoantennas with the dimensions of length *L* = 1.33 um and width *W* = 0.24 um were uniformly arrayed with period *P* = 1.6 um. Both the cross-shaped Au nanoantenna and MgF_2_ spacer were offset from the center with the asymmetric parameter *s* = 0.265 um, which can help support dual-band resonance for the detection of two kinds of molecules simultaneously. In addition, the arm lengths of the MgF2 dielectric spacer were Δ*l* smaller than those of the Au nanoantenna, which enabled more sensing area exposure to the analyte molecules. Figure 1b shows the side view of three-layered the structure. The thickness of the top Au layer *h* and MgF_2_ spacer *t* were 70 nm and 90 nm, respectively. The Au substrate with a thickness of 200 nm ensured that there was no light transmission. Our DBPA structure was confirmed feasible in the experiment, with the guide for detailed fabrication procedures in reference [15].

For identifying and quantifying composite vibrational fingerprints of multiple biomolecules, we used the protein and lipid molecules as the target molecules, which were spread on our DBPA and almost overlapped with hot spots. Lipid molecules have two infrared fingerprint vibrations located around wavelengths of 3427 and 3509 nm [15], and protein molecules located around 6020 nm and 6410 nm [24]. Besides proteins and lipids, other biochemicals such as peptides, sugars, and nucleic acids can also be detected with our DBPA as long as the plasmonic resonance is tuned to close to the vibrational band of the molecules [25].

The finite element method was applied to calculate the optical response of the DBPA by using the software COMSOL Multiphysics 5.6. A TM plane wave impinged on the Au nanostructure with an incident angle of *θ*. Periodic boundary conditions along the *x*- and *y*-directions were applied to mimic the infinite planar array. Perfectly matched layers were set along the z-direction to avoid unphysical reflections. The dielectric permittivity of Au was taken from Ref. [15]. The permittivity of MgF_2_ was described by a constant *ε*_M_ = 1.382. Both lipid and protein molecules contain two vibrational bands. Lipid molecules include CH_2_ and CH_3_, and protein molecules contain amide I and II bands, which are primarily associated with the C=O stretch and N-H bend modes in the amide functional group. The absorption of these vibrational bands was nearly a Lorentizian line shape; thus, the molecular permittivity could be fitted to the Lorentizian model $\varepsilon =n_{\infty }^{2}+\sum_{k=1}^{2}\frac{A_{k}\omega_{k}^{2}}{\omega_{k}^{2}-\omega_{k}^{2}-i\omega \gamma_{k}}$. Next, the analytic model was used to retrieve the molecule’s permittivity from the experimental results by adjusting a Lorentzian permittivity [24]. Good agreement was observed between the experimental and calculated spectra for both the protein and lipid molecules. The molecules introduced not only the vibrational resonances (the second term) but also the increased effective index $n_{\infty }^{2}$ (the first term). We assumed the dielectric non-dispersive background $n_{\infty }^{2}=2.36$. *k* = 1, 2 corresponded with the contributions from two vibration bands of the molecules. When it came to the lipid molecules, it had parameters with *ω*_1_ = 2930 cm^−1^, *ω*_2_ = 2850 cm^−1^, *γ*_1_ = 13.3 cm^−1^, *γ*_2_ = 9.56 cm^−1^, *A*_1_ = 0.0048, and *A*_2_ = 0.0048, respectively. The two vibrational fingerprints of protein molecules had parameters with *ω*_1_ = 1668 cm^−1^, *ω*_2_ = 1532 cm^−1^, *γ*_1_= 78.1 cm^−1^, *γ*_2_ = 101 cm^−1^, *A*_1_ = 0.016, and *A*_2_ = 0.017, respectively. These parameters were from the works of Refs. [14,25]. The permittivity of the mixed molecules can be described as $\varepsilon =\varepsilon_{L}+\varepsilon_{P}$, where *ε*_L_ and *ε*_P_ are the permittivity of the lipid and protein molecules, respectively.

Classic oscillator model also can be utilized to study the strong multimode coupling behavior under study [26,27]:

(1) $$\left(\begin{array}{ccc} E_{p} & \hbar \Omega_{1}/2 & \hbar \Omega_{2}/2\\ \hbar \Omega_{1}/2 & E_{1} & 0\\ \hbar \Omega_{2}/2 & 0 & E_{2} \end{array}\right)\left(\begin{array}{c} \alpha_{p}\\ \alpha_{1}\\ \alpha_{2} \end{array}\right)=E\left(\begin{array}{c} \alpha_{p}\\ \alpha_{1}\\ \alpha_{2} \end{array}\right)$$

where *E*_p_ is the energy of the original plasmon mode, and *E*_1_ and *E*_2_ represent the energy of the two vibrational bands of the molecules. *E* is the eigenvalue corresponding to the energies of the hybrid modes after coupling. |*α*_p_|^2^, |*α*_1_|^2^, and |*α*_2_|^2^ represent the weighting coefficients of the original modes in the hybrid modes (|*α*_p_|^2^ + |*α*_1_|^2^ + |*α*_2_|^2^ = 1). *ħ*Ω_1_ and *ħ*Ω_2_ are the coupling energy between the plasmon and molecules modes. Usually, the two vibrations of molecules cannot couple; thus, the coupling energy between *E*_1_ and *E*_2_ is zero.

## 3. Results and Discussion

Figure 1c shows the absorption spectra of the bare DBPA (the dashed line), pure molecules without the DBPA (the dotted line), and DBPA-molecule interaction system (the solid line), respectively. Here, the absorption is defined as *A* = 1–*R*–*T*, where *R* is the reflection and *T* is the transmission. The thickness of the gold substrate is much larger than the skin depth of the gold, which makes the transmission *T* = 0, and the absorption can be simplified as *A* = 1 − *R*. The bare DBPA supports two plasmonic modes with perfect absorption in the absorption spectrum, which are labeled as *P*_1_ and *P*_2_. Both of the plasmon modes *P*_1_ and *P*_2_ arise from near-field coupling between the two which generates antiparallel currents at the antenna and the metal film surface. The plasmon-resonant wavelength obeys the role $\lambda =2L_{eff}n_{eff}/m+C$ [12,13,14], where *L_eff_* is the effective length of the arm, *n_eff_* is the effective refractive index of the surrounding medium, *C* is a constant, and mode number *m* is equal to one here for the first-order mode. For the *x*-direction light polarized, our asymmetric unit cell in the *x*-direction gives rise to two effective arm lengths with $L_{1}\approx L/2-s$ and $L_{2}\approx L/2+s$, therefore, providing the corresponding dual-band absorption. The mode *P*_1_ at a shorter wavelength (3450 nm) is associated with the shorter antenna *L*_1_ while the other mode *P*_2_ at a longer wavelength (6280 nm) is associated with the longer antenna *L*_2_. In addition, the absorption of the two kinds of molecules without the aid of DBPA is also shown to provide the corresponding information of vibrational fingerprint signals. Many absorption features are present in this spectrum, notably four absorption bands due to the vibrational modes of the mixed molecules. The former two absorptions at 3427 nm and 3509 nm originate from the lipid molecules, whereas the latter two of interest at 6020 nm and 6410 nm originate from the protein molecules. It is shown that the absorption signal is so weak without coupling with the plasmon mode yet. It is also worth noting that the absorption spectrum of the uncoupled DBPA here already takes the dielectric non-dispersive background $n_{\infty }^{2}=2.36$ of the molecule’s permittivity into account. The dimensions of DBPA are carefully designed so that the resonance wavelengths of *P*_1_ and *P*_2_ spectrally match up with the spectral regions of the target biomolecules for the preparation of the following coupling. The absorption of the DBPA-molecule coupled system displays distinct dips on both *P*_1_ and *P*_2_ resonance, which indicates the enhanced lipid and protein molecular vibrational signals.

**Figure 2:**
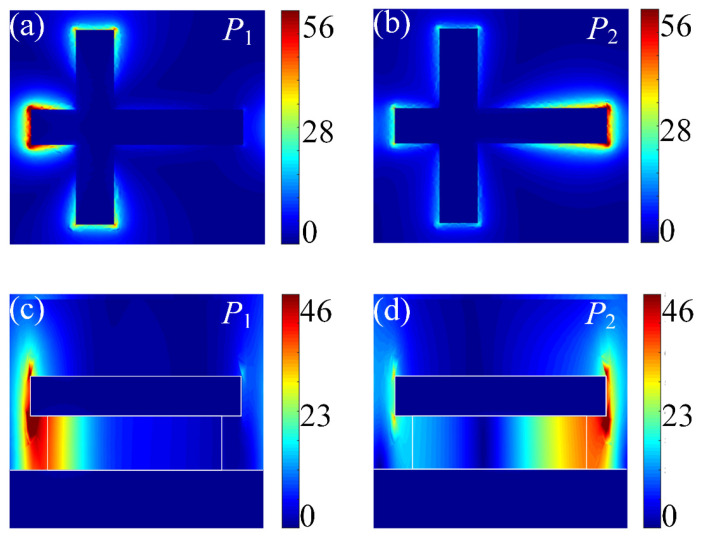
Electric field distributions (|*E*|/|*E*₀|) at (**a**) *P*_1_ (3450 nm) resonance and (**b**) *P*_2_ (6280 nm) resonance (top view), indicating the excitation of shorter antenna and longer antenna, respectively. Cross-sectional views at *P*_1_ (**c**) and *P*_2_ (**d**) modes show enhanced fields extending into the surrounding region, ensuring molecules’ access to the plasmonic enhanced field. The color gradients represent enhanced field intensity (blue: low; red: high).

Physically, the coupling strength between molecules and the plasmon of DBPA mainly depends on the electric field intensity of the DBPA. Figure 2 shows the simulated near-field distribution of the two modes *P*_1_ and *P*_2_ at the respective resonance wavelengths. In particular, Figure 2a,b are the top views of the structure, which is monitored beneath the top Au cross antenna. As expected, highly enhanced local electric fields are mainly concentrated around the end of the shorter antenna at *P*_1_ resonance and a similar enhancing effect is observed around the other end of the longer antenna at *P*_2_ resonance. The cross-sectional view of field intensity distribution is reported in Figure 2c,d. The plot proves again that the local near-field enhancement is concentrated at the upper and bottom end of the corners of the top cross nanoantenna. Moreover, the maximum enhancing field at the bottom end keeps extending down widely into the surrounding environment. Benefiting from that, the middle dielectric spacer has a size smaller than the top Au cross antenna, and the analyte molecules have the chance to access the remaining space between the top antenna and the substrate, where the electric field enhancement is accessible. Considering the above two points, an increased effective sensing area is obtained, which results in a stronger mode coupling between the vibrations of the analyte molecules and the plasmonic mode of the DBPA structure.

**Figure 3:**
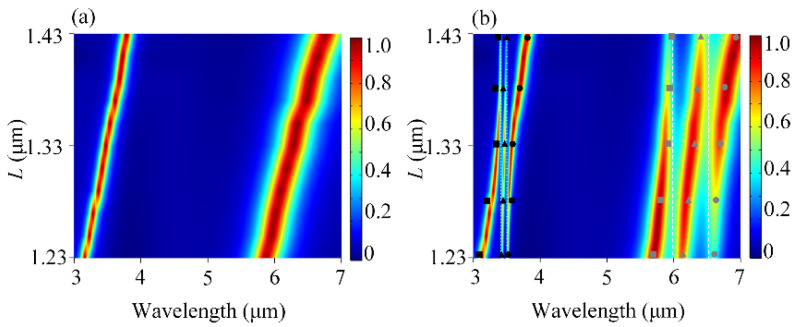
(**a**) Contour plot of the absorption spectra of the uncoupled DBPA as a function of top antenna length *L*. (**b**). Contour plot of the absorption spectra of the coupling behavior between DBPA and the mixing molecules as a function of *L*. The color gradients represent absorption intensity (blue: low; red: high). The scatters (squares, triangles, and squares) stand for the three new hybrid polaritons based on the oscillator model.

Then, we systematically investigated the coupling between the mixed molecules and the plasmonic modes in the DBPA structure. Figure 3a shows the contour plot of the absorption spectra of the uncoupled DBPA as the function of the top antenna length *L*. Here, the other structural parameters are the same as before. One can see that both the resonance wavelengths of two modes *P*_1_ and *P*_2_ exhibit a red shift and the absorptions are almost perfect when the *L*-value increases from 1.23 to 1.43 μm. The increasing *L* is equal to the simultaneous increase in *L*_1_ and *L*_2_. For mode *P*_1_, it shifts from 3.16 to 3.79 μm, moving across the two vibrational modes of lipid molecules (~3427 nm and ~3509 nm) one after another. For mode *P*_2_, it red-shifts from 5.86 to 6.76 μm, running across the two vibrational modes of protein molecules (~6020 nm and ~6410 nm) successively. After adding the mixing molecules, Figure 3b plots the absorption of the DBPA-molecule coupled structure. We first took a look at the change in *P*_1_ mode. It can be seen that the *P*_1_ band is split into three resonant peaks and two valleys, accompanied by double anti-crossing behavior. This is known as the Rabi splitting, typical for strongly interacting multilevel systems [28], and caused here by the strong coupling of plasmon resonance *P*_1_ with two vibrational resonances in lipid molecules. Rabi splitting can also be defined by $\Omega_{Rabi}=2\sqrt{g^{2}-{\left(\Gamma_{p}-\Gamma_{m}\right)}^{2}/16}$ [29], where Γ*_p_* and Γ*_m_* are the full-width at half-maximum (FWHM) of the uncoupled plasmon and molecular resonance, and *g* is the coupling strength. At *L* = 1.33 μm, the double anti-crossing behavior is uniform with the same Rabi splitting values *ħ*Ω of 11.3 meV. The evident two dips reveal the presence of the vibrational modes in lipid molecules. The same phenomenon also exists in the coupling between *P*_2_ and two vibrational resonances in protein molecules. The two splitting *ħ*Ω also share the same values of 12.8 meV at *L* = 1.33 μm and the two dips correspond to the position of two vibrational resonances in the protein molecules. We also applied the three-oscillator model to study the strong multimode coupling behaviors [26,27]. Both the *P*_1_ and *P*_2_ modes couple with two vibrational modes of lipid and protein molecules, respectively, which can be seen as two sets of three-oscillator models. In each set, scatters (squares, triangles, and squares) represent the calculated eigenvalues *E* from Equation (1) by taking *E*_p_, *E*_1_, *E*_2_, *ħ*Ω_1_, and *ħ*Ω_2_ into account, corresponding to the energies of the newly formed hybrid modes. There is a good match between the theoretical calculations and simulation results [27]. By the strong coupling with DBPA structure, the characteristic vibrational fingerprints of molecules are amplified and become obvious to be detected. Additionally, two well-separated spectral locations can enable the simultaneous identification of composite vibrational fingerprints in multiple biomolecules. Moreover, it is noted that the length of *L*_1_ and *L*_2_ can be modified independently, which provides flexible tunability for the plasmonic resonance to the desired wavelength. Such independence is a key characteristic that allows the DBPA structure to be adjusted in a simple manner to enhance the vibrational bands of different analytes.

**Figure 4:**
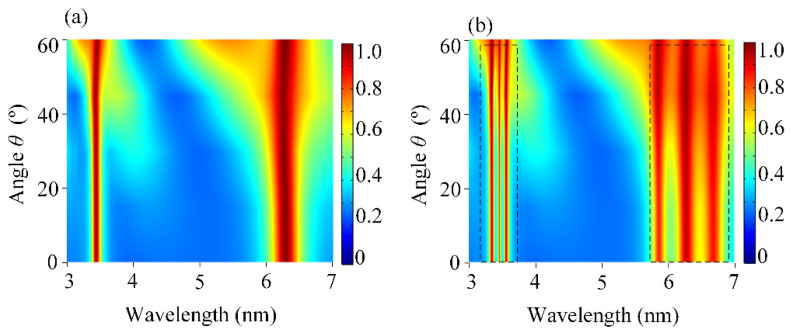
(**a**) Contour plot of the absorption spectra of the uncoupled DBPA as a function of the incident angle *θ*. (**b**). Contour plot of the absorption spectra of the coupling behavior between DBPA and the mixing molecules as a function of *θ*. The color gradients represent absorption intensity (blue: low; red: high). The dashed boxes represent the enhanced signal of lipid and protein molecules, respectively.

It is well known that the insensitivity to the incident angle for the device is very important in practical applications [30]. Therefore, we also explored the effect of the incident angle *θ* on the absorption of DBPA and the coupling phenomenon under different *θ*. Under TM polarization, Figure 4a shows the contour plot of the absorption of DBPA as a function of incident angle *θ*. When the angle of incidence was up to 60°, the perfect absorption of *P*_1_ and *P*_2_ remained very high while maintaining the position of the center wavelength unchanged. Taking the molecules into account, the molecular signals are depicted distinctly, as shown in Figure 4b. When we matched the plasmonic resonance band with the molecular absorption band, the molecules’ vibrational bands started to absorb more energy from the DBPA, which offered the enhanced field available. When *θ* = 0°, the maximum sensitivity was achieved, as well as the most enhanced molecular signal. This arose from the symmetric electric field distribution generated at *θ* = 0°, which maximized the spatial overlap between the enhanced fields and target molecules. At normal incidence, the absorption of the *P*_1_ and *P*_2_ resonances exceeded 96%, accompanied by maximal Rabi splitting depths. With the increasing *θ*, both absorption bands still appear as two pronounced dips, which corresponded to the vibrational bands of the lipid and protein molecules, respectively. Additionally, these dips were found to be resistant to angles as well, which could present molecular signals clearly without the perturbed position. It was essentially demonstrated that these absorption bands depend weakly on the angle of incidence. For oblique angles, while the plasmon–molecule coupling strength gradually weakened due to reduced field localization, the absorption remained high (>90% up to *θ* = 60°) and the molecular signals remained evident. As our simulation results reveal, the designed DBPA can operate well over a wide range of incident angles for both absorption peaks. The availability of wide-angle incidence is quite useful for detecting the characteristic vibrational bands of molecules.

**Figure 5:**
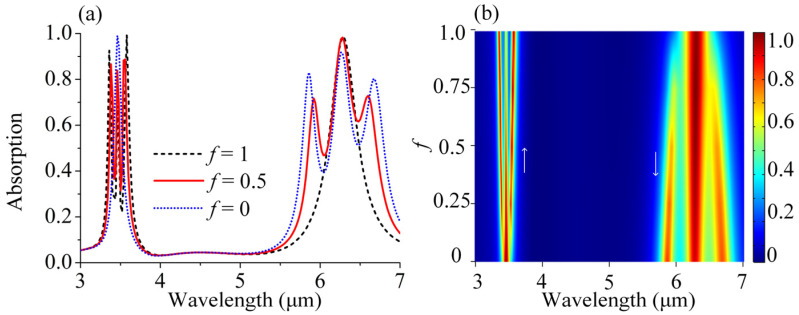
(**a**) Absorption spectra of the coupled behavior between DBPA and the mixing molecules with different mixing ratios *f*. (**b**) Contour plot of the absorption spectra of the coupling behavior between DBPA and the mixing molecules as a function of *f*.

For the mixed solutions, it is significant not only to identify the presence of multiple molecules, but also to quantify the concentration ratio of the corresponding molecules. We next analyzed the optical response of the mixed lipid and protein molecules with varying mixing ratios in Figure 5. Here, the total amount of lipid and protein molecules remains the same, and *f* represents the ratio of the lipid molecules. In simulation, the permittivity of the mixed molecules *ε*_m_ can be described by *ε*_m_ = *fε*_L_ + (1 − *f*)*ε*_P_. In this case, the real part of *ε*_m_ stayed the same, without any additional impact on the position of *P*_1_ and *P*_2_. The arm length *L* and the overall amount of the mixed molecules were kept constant to ensure that the resonance position of the *P*_1_ and *P*_2_ modes nicely overlapped with the two vibrational bands; thus, the information of different concentrations could be read directly by the Rabi splitting depth of the *P*_1_ and *P*_2_ modes. Firstly, we took three simple sets of data for example, which started from a pure lipid molecules solution (*f* = 1), followed by an equal mixture of lipid and protein molecules (*f* = 0.5), and ended with a pure protein molecules solution (*f* = 0). Inspecting the spectra closely, the concentration variation in the mixtures gave different modulation depths and Rabi splitting. For *f* = 1 (black curve), there were no protein molecules in the analyte and only pure lipid molecules instead, and a higher concentration of lipid molecules led to stronger coupling and larger Rabi splitting. Consequently, a more pronounced vibration feature was presented in the *P*_1_ mode, whereas the *P*_2_ mode showed no dips. Similarly, for *f* = 0 (blue curve), there were only protein molecules instead and there are two pronounced spectral dips in the *P*_2_ mode, whereas the *P*_1_ mode shows nearly no change. For *f* = 0.5 (red curve), there were equal amounts of protein molecules and lipid molecules in the analyte, which were half the concentration of the pure analyte when *f* = 0 and *f* = 1. Both the *P*_1_ and *P*_2_ modes showed dips at molecular vibrations, which were slightly gentle compared to those in the pure analyte of 100% concentration. To show the variation trend clearly, we gave more general cases and converted these absorption data to a two-dimensional *f*-resolved map by varying the mixing ratios of lipid molecules from 0 to 1. As shown in Figure 5b, it is obvious that both the coupling modulation depth and the Rabi-splitting energy varied with the concentration, and a higher respective concentration led to a larger modulation and a wider Rabi-splitting, which represented a more pronounced vibration feature. By utilizing DBPA, not only the different pure solutions but also the different mixture ratios can be clearly distinguished.

As mentioned before, the Rabi-splitting depth can act as a good indicator to identify the analytes with varying mixing ratios *f*. However, this convenience is based on the condition that the resonance of plasmon modes overlaps nicely with vibrational bands of molecules. We know that the molecules will introduce not only the vibrational resonances but also the increased background refractive index, which causes a shift in the plasmon resonance. Only if the total quantity of molecules is chosen carefully does the resonance of plasmon modes shift to the desirable wavelength to match the vibrational resonances of molecules, as discussed above. Generally, the total amounts of molecules may be more or less than the standard value in practical applications, in which case the information of analytes may not be apparent to the eye due to the detuning between the resonances of plasmon modes and vibrational modes. Here, we introduced a mathematical algorithm known as principal component analysis (PCA) for state-of-the-art data analysis, which can convert complex absorption spectra into simple information [23]. PCA algorithm is a technique for simplifying data sets and is mainly used for dimension reduction, which is to project the sample data from high-dimensional space to low-dimensional space and represent the original data in low-dimensional space as much as possible. Mathematically speaking, it uses the eigenvector-eigenvalue principle. Its goal is to extract important information from the original data set to represent it as a set of new orthogonal eigenfunctions called principal components (PCs) and eigenvalues which are termed scores (SCs). In our case, there were 10 sets of original data in total, which were composed of 5 sets whose total amount of mixed molecules was less than the standard value (80:0), (60:20), (40:40), (20:60), and (0:80), and the other 5 sets with total amount of mixed molecules more than the standard value (120:0), (90:30), (60:60), (30:90), and (0:120). In each absorption spectrum, the wavelength value increased from 3000 nm to 7000 nm with the step of 20 nm; thus, there were 201 absorption data points in each set and the original data could be described by the 10 × 201 matrix ***X***. Here, we expected to reduce ***X*** to two dimensions matrix *X*’ (10 × 2) and these ten complex absorption spectra could be simplified into ten points. PCA decomposes the original data ***X*** and represents it with the form of eigenfunctions (PCs) and eigenvalues (SCs), which can be described as follows:

***X*** = SC·PC + *u* (2)

Here, *u* is the average of data in matrix *X*. The utilized algorithm determines the PCs such that the first one contributes the highest variance and thus constitutes the largest contribution. Analogously, the second-order PC makes the second largest contribution, and so forth. The correlation between individual data sets is more significant when fewer PCs are needed in order to describe the entire data set. We utilized the built-in pca function in MATLAB 2022 to perform PCA on the spectral data set, which operates automatically the data standardization, covariance matrix calculation, eigenvalue decomposition, principal component selection, and data transformation, optimizing computational efficiency and numerical stability, particularly for large data sets.

**Figure 6:**
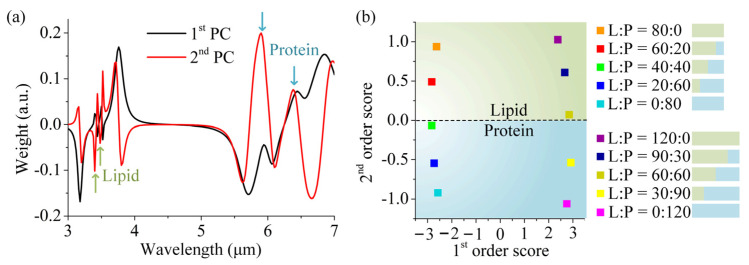
PCA of mixed lipid and protein molecules: (**a**) The first and second principal components as obtained from PCA. (**b**) Plotting the corresponding second-order score vs. first-order score for each measurement reveals clusters for each measurement step. The characteristic positions obtained from the input parameter independent algorithm can be addressed to specific concentrations by calibration measurements.

Figure 6a shows the first two PCs of our data. It is important to note that the PCs, as calculated by the algorithm, have no a priori physical interpretation [23]. The corresponding first- and second-order SCs are depicted in Figure 6b. As a result of the dimension reduction process, the original ten sets of spectral data are simplified and well encoded into ten points of data, which automatically forms distinct and clear zoning. The different kinds of mixed molecules as well as their respective concentrations can be clearly separated and identified in the 2D space of the first- and second-order scores. Firstly, we noticed that ten data points were distributed into two regions at two sides of the x-axis, respectively. The five points on the left-hand side (*x* < 0) share the same total amount of mixed molecules, which is lower than the standard value. Also, they share the nearly same x-value. The same behavior also exists for the five points on the right-hand side (*x* > 0), whose molecular total amount are more than the standard value. It is reasonable to infer that the first SCs reflect the total number of the mixed molecules: the larger the x-value, the more mixed molecules, and vice versa. Additionally, the *x* = 0 is the position for the standard value of the total amount, acting as a reference. The first SCs with the same total amount are not able to distinguish the concentration ratio of the particular molecules, which can be solved with the assistance of the second SCs. We can see that the second SCs can differentiate the lipid molecules from protein molecules in the mixed analytes with the same total amount, and quantify their respective concentration. The second SCs also divide the data into two regions, where the green region (*y* > 0) represents the dominance of lipid molecules, while the blue region (*y* < 0) means the majority of lipid molecules. Accordingly, the dashed line (*y* = 0) is the medium line, around which the points have equal concentrations of lipid and protein molecules in the analytes. Interestingly, it is also found that the identical relative concentration ratios almost share the same *y*-value, such as the set (60:20) and (90:30). Now, we can assign PCs in Figure 6a as a physical interpretation that applies to our case. The first PC takes the form of the resonances that roughly correspond with the plasmonic resonance of the DBPA. When adding or subtracting the molecular amount from the standard value of the total amount, the corresponding plasmon mode is blue-shifted red-shifted, which arises from its sensitivity to the external effective refractive of molecules. The second PC is found to contain the vibrational information. As indicated by the green and blue arrows, all four vibrational signatures are encoded in the second PC. It is noted that the vibrational bands of lipid molecules and protein molecules have opposite directions, and thus, the sign of the second score is distributed at the two sides of the y-axis. The position of 10 data points from the PCA algorithm shows a good match to the physical interpretation, and the gradation of color is further used to represent the absolute concentration of each molecule for simplicity. This convenience can be extended to more general situations, and PCA still works well, free of solvent interference. The versatility of PCA manifests in two critical aspects: Firstly, in noise suppression, by retaining only dominant principal components, minor fluctuations induced by stochastic noise or measurement uncertainties can be filtered out, significantly enhancing the signal–noise ratio. Secondly, even in non-ideal conditions where plasmonic resonances and molecular resonances are spectrally misaligned due to refractive index variations or concentration changes caused by biomolecules, PCA can decouple spectral data from complex absorption spectra, reducing high-dimensional information into distinct features that reveal molecular composition and concentration in a simplified manner. More importantly, when the concentration is reduced to 10% of the original value (e.g., from 80 nm to 8 nm for lipid–protein mixtures), our DBPA sensor still works, which can identify the molecular signals. When at an ultra-low concentration of 1%, though molecular information cannot be distinguished by the absorption spectra, PCA still helps the identification and quantitation of the mixed molecules. Thus, by the combination of our sensor and PCA, we can achieve excellent sensitivity at ultra-low concentration.

## 4. Conclusions

In summary, this study presents an advancement in mid-infrared molecular sensing by integrating a dual-band perfect absorber (DBPA) plasmonic sensor with machine learning algorithms for the simultaneous qualitative and quantitative detection of multiple biomolecules. This innovative sensor design enables more sensing area exposed to the analyte molecules and gives rise to stronger coupling. To address challenges in quantifying molecular concentration under ratio variations, a principal component analysis (PCA) algorithm is introduced. PCA effectively decouples spectral data, enabling the precise identification of molecular concentrations even in non-ideal conditions. This combination of plasmon-enhanced sensing and machine learning establishes a versatile, noninvasive platform for detecting complex biomolecular mixtures. Overall, this work opens new avenues for optical sensing in fields such as biomedical diagnostics, environmental monitoring, and chemical analysis, offering enhanced sensitivity, multiplexed detection, and autonomous data interpretation. By leveraging the flexibility of our sensor and the robust signal processing power of PCA, we are confident that the sensor can be adapted to a wide range of biological molecules and complex samples in the real world. We look forward to providing experimental evidence in future work.

## Data Availability

The data of our study are available upon request.
